# The Effect of Aerobic Exercise on Occupational Stress of Female Nurses: A Controlled Clinical Trial

**DOI:** 10.17533/udea.iee.v37n2e05

**Published:** 2019-09-19

**Authors:** Zinat Mohebbi, Setareh Fazel Dehkordi, Farkhondeh Sharif, Ebrahim Banitalebi

**Affiliations:** 1 Ph.D. School of Nursing, Shiraz University of Medical Sciences, Shiraz, Iran. Email: mohebbi04@yahoo.com. Corresponding author. Shiraz University of Medical Sciences Shiraz Iran mohebbi04@yahoo.com; 2 M.Sc. School of Nursing, Student Research Committee, Shiraz University of Medical Sciences, Shiraz, Iran. Shiraz University of Medical Sciences Shiraz Iran; 3 Ph.D. Community base Psychiatric Care Research Center, Shiraz University of Medical Scienses, Shiraz, Iran. Shiraz University of Medical Scienses Shiraz Iran; 4 Ph.D. University of Shahrekord, Shahrekord, Iran. Shahrekord University University of Shahrekord Shahrekord Iran

**Keywords:** control groups, physical exertion, occupational stress, nurses, female., grupos control, esfuerzo físico, estrés laboral, enfermeros, femenino., grupos controle, esforço físico, estresse ocupaciona, enfermeiras e enfermeiros, feminino.

## Abstract

**Objective.:**

This work sought to determine the effectiveness of an aerobic exercise program on the occupational stress of nurses.

**Methods.:**

Prevention-type controlled clinical trial carried out with the participation of 60 nurses working in hospitals affiliated to Shahrekord University of Medical Sciences in Iran. Randomly, the nurses were assigned to the experimental group or to the control group. The intervention consisted in an aerobic exercise program lasting three months with three weekly sessions one hour each. The Health and Safety Executive (HSE) questionnaire measured occupational stress with 35 questions, each with five Likert-type response options, which can have a maximum score of 175 points; higher scores meant lower levels of occupational stress. The HSE was evaluated during three moments: upon registering, after finishing the exercise program (week 8), and two months after terminating the intervention (week 16).

**Results.:**

The level of occupational stress was the same in the experimental and control groups during registration (86.2 vs. 86.3). Upon finishing the aerobic exercise program (week 8), the experimental group showed a higher score than the control group (119.7 vs. 86.2, *p*<0.01), with this score diminishing after two months of having ended the intervention (91.4 vs. 85.8, *p*=0.061).

**Conclusion.:**

The aerobic exercise program was associated to decreased work stress of nurses in the experimental group compared to the control group at eight weeks, but this difference did not persist when the experimental group did not continue with the program.

## Resumen

**Objetivo.:**

Determinar la efectividad de un programa de ejercicio aeróbico en el estrés ocupacional de las enfermeras.

**Métodos.:**

Ensayo clínico controlado de tipo preventivo que se llevó a cabo con la participación de 60 enfermeras que trabajaban en hospitales afiliados a *Shahrekord University of Medical Sciences* en Irán. Las enfermeras se asignaron aleatoriamente al grupo experimental o al grupo control. La intervención consistió en un programa de ejercicio aeróbico realizado durante dos meses con tres sesiones semanales de una hora de duración. El estrés ocupacional se midió con el cuestionario *Health and Safety Executive* (HSE) de 35 preguntas, con opciones de respuesta tipo Likert que van de 1 a 5; puede llegar a puntuar en 175 como máximo: a mayor puntaje, menor el nivel de estrés ocupacional. Se evaluó el HSE en tres momentos: en la inscripción, después de finalizar el programa de ejercicio (semana 8) y también dos meses después de terminada la intervención (semana 16).

**Resultados.:**

Fue igual el nivel de estrés ocupacional en los grupos de intervención y de control en la inscripción (86.2 vs. 86.3), pero al finalizar el programa de ejercicios aeróbicos (semana 8) el grupo experimental mostró un puntaje mayor que el del grupo control (119.7 vs. 86.2, *p*<0.01), para luego disminuir este puntaje a los dos meses de haber finalizado la intervención, (91.4 vs. 85.8, *p*=0.061).

**Conclusión.:**

El programa de ejercicio aeróbico se asoció a la disminución del estrés laboral de las enfermeras en el grupo de intervención en comparación con el grupo control a las ocho semanas, pero esta diferencia no se mantuvo cuando el grupo experimental no continuó con el programa.

## Resumo

**Objetivo.:**

Determinar a efetividade de um programa de exercício aeróbico sobre o estresse ocupacional das enfermeiras.

**Métodos.:**

Ensaio clínico controlado de tipo preventivo que se levou a cabo com a participação de 60 enfermeiras que trabalhavam em hospitais afiliados a Shahrekord University of Medical Sciences no Irã. Em forma aleatorizada, as enfermeiras foram designadas ao grupo experimental ou ao grupo controle. A intervenção consistiu num programa de exercício aeróbico realizado durante dois meses com três sessões semanais de uma hora de duração. O estresse ocupacional se mediu com o questionário Health and Safety Executive (HSE) de 35 perguntas, que tem opções de resposta tipo Likert que vão de 1 a 5, pode chegar a pontuação de 175 como máximo; a maior pontuação, é menor o nível de estresse ocupacional. Se avaliou o HSE em três momentos: a inscrição, depois de finalizar o programa de exercício (semana 8) e também dois meses depois de terminada a intervenção (semana 16).

**Resultados.:**

Foi igual o nível de estresse ocupacional nos grupos de intervenção e de controle na inscrição (86.2 vs. 86.3), mas ao finalizar o programa de exercícios aeróbicos (semana 8) o grupo experimental mostrou uma pontuação maior que o do grupo controle (119.7 vs. 86.2, *p*<0.01), para depois diminuir esta pontuação aos dois meses de haver finalizado a intervenção, (91.4 vs. 85.8, *p*=0.061).

**Conclusão.:**

O programa de exercício aeróbico se associou à diminuição do estresse profissional das enfermeiras no grupo de intervenção em comparação com o grupo de controle às oito semanas, mas esta diferença não se manteve quando o grupo experimental não continuou com o programa.

## Introduction

In psychology, stress or psychological pressure means pressure, and force and any motivation that produces stress in human beings is called stressor or stressor factor. Rice(1) suggests that stress is the non-specific reaction of the body against any request; he points out that the objective of non-specific reactions is creation of physiologic equilibrium and adaptation. Stress leads to chronic diseases, like hypertension, cardio-vascular diseases, asthma, etc.,(2) Stress also affects the health of individuals, reduces quality of life, and increases the probable incidence of job-related injuries.(3)

Nowadays, occupational stress has become a prevalent problem in workplaces and few people have not faced such problems. The Princeton Survey Research Institute indicated that 75% of workers suffered from occupational stress compared with the past generation.(4) Occupational stress occurs when a person’s expectations are more than his/her abilities.(5) Several occupational stressor factors exist, which could be divided into two groups of intra- and extra-organization. Factors related to the type of occupation, like workload, and group factors, such as lack of group support, are among the intra-organization factors and economic undesirable conditions and family environment are among the extra-organization factors.(6) Severe stress in the workplace causes much damage and expense to individuals, as well as organizations; occupational stress threatens the health of employees who are effective in productivity.(7) Currently, health and treatment are considered among the most important domains of permanent development of human societies and because they have a direct relationship with human health, healthy and lively nurses are needed.(8) According to the results from a research(9) on the effects of stressors in the workplace on nurses, 93% of the participants were regularly affected by the stressor factors of work environment. Among health staff, the nursing profession has been known as one of the occupations with high risk from the vantage point of fatigue and disease and the hospital environment can lead to stress and physical problems among nurses.

A study has reported that stress among nurses is at 42%,(10) given the occupational nature of the nurses who have a direct relationship with patients and their care, lack of awareness and exposure to such stress will result in some irreparable complications. The continuity or severity of stressor factors could cause occupational burnout among nurses. Empowering nurses to confront stressor factors, along with producing some conditions to reduce and eliminate such factors, could play an effective role in creating a peaceful working environment and increasing the capacity and efficiency of nurses.(6) A study with 1500 nurses working in 31 health centers in England(11) and who had applied for long leave considering considerable occupational stress, which was at 27%. Other nurses have suffered from depression and aggression after such conditions. A government census in England showed that the cause of 30% of leaves in health centers in this country was occupational stress with yearly expenses > 400-million pounds, followed by loss of profitability and replacement of staff in these centers. Leaves and burnout due to occupational stress for nurses in Scotland and Thailand increased significantly in 2015. In research carried out in 2012 and 2013, about 38.0% of the staff in health centers reported suffering from severe stress. According to said research, 7.4% of the nurses were absent weekly due to occupational fatigue or stress-related inability, which is about 80.0% more than other occupational groups.(9) In Iran, the 2013-2015 census showed that more than one third of nurses suffered from poor mental health.(12) Some activities, like seeking help from others to do the work, looking forward to support from others; being realist; considering the situations; doing exercise; having an appropriate diet; getting enough sleep and rest; enjoying healthy recreation; taking trips; laughing; writing one’s own thoughts and feelings; and having self-confidence are among the methods of coping with stress.(13)

According to Brunner and Suddarth, various methods exist for adaptation and each individual applies a specific method. These methods include relaxation, communication, deviation of senses, exercise, sufficient rest, eating, drinking, etc.(14) For this reason, using simple methods with no complication, like exercise, seems to play an important role in reducing stress. Currently, people look at exercise and physical activities not only as ways of spending leisure time, but also as an undeniable necessity for health.(13) The positive impacts of exercise and regular physical activities have been confirmed in several investigations with children, adolescents, youth, adults, and even the elderly.(15) Aerobic exercise is a series of muscular periodic and rhythmic movements, which increase respiratory and heart rate at a particular time.(16) Guszkowska(17) carried out a study on the effect of aerobic exercise on anxiety and depression and showed that exercise is effective in reducing anxiety and depression after 10 sessions and the anti-depression and anti-anxiety effects of exercise continue for one month after treatment. Another research also showed that physical activity can play a positive role in securing and providing mental health and is effective in reducing anxiety and depression, increasing mental health, and promoting quality of life.(18) 

Some researchers have shown that exercise has a significant effect in reducing stress,(19) but some other studies, like that conducted by Sorensen *et al*.,(20) showed opposite results. Their study on the evaluation of the effect of exercise on police officers reported that no significant relationship existed between increased physical activity and reduced stress. Anyhow, no agreement exists indicating that perseverance and aerobic ability of the body and muscles can protect the individual against various stressors related to lifestyle or occupation. As mentioned, stress affects adversely the mental health of nurses. Considering the unavoidability of some stress factors in the nursing profession and the need to prevent the effects of physical and behavioral stress, taking some measures to improve the quality of life and teaching some coping strategies are among the responsibilities of managers to prevent their burnout and migration.

By reviewing databases, like Scopus and Pubmed, we found that no research exists on the effects of aerobic exercise on occupational stress of female nurses. In addition, most research has focused on studying the rate and reasons of stress among nurses(7) and interventions conducted to reduce their stress have mostly been educational workshops of short duration, with few interventions involving exercise programs.(19) Researchers sought to determine if a selected aerobic exercise program could play any role in reducing the stress of nurses, so that, by using these data, health-treatment planners and managers prevent the adverse effects of stress among nurses and increased costs of its non-observance. Therefore, the present research studied the effect of aerobic exercise on the amount of stress of nurses.

## Methods

This was an interventional and clinical trial study. The population included 60 nurses working in the hospitals affiliated to Shahrekord University of Medical Sciences in Shahrekord-Iran; they were selected purposefully and divided into two experimental and control groups after completing the questionnaire. The method used to categorize the two groups was block randomization with a size of four.

Inclusion criteria consisted of having at least one year of work experience, suffering occupational stress considering the HSE (Health and Safety Executive) occupational stress questionnaire, age limitation of 25 - 40 years, informed consent, along with non-existence of any type of disease, ability of engaging in exercise activity, lack of participation in any organized physical activity during the last two months and during research, and not enduring any severe stress during the previous six months. Exclusion criteria involved being absent for more than three continuous exercise sessions, being absent for more than six sessions during exercise sessions, and suffering unpleasant events (severe stressor accident) by the participant during the exercise sessions. Demographic information of the samples showed that demographic variables of the two control and experimental groups did not have any significant statistical difference. 

The Health and Safety Executive (HSE) is a questionnaire used to determine occupational stress. This questionnaire includes 35 questions in seven domains (demand, control, responsible supports, colleague support, relaxation, role, and changes) and uses a 5-point Likert scale (never, seldom, sometimes, mostly, and always). Scoring is inverse so that high scores indicate higher health and security from the stress point of view and low scores show lower occupational stress. The reliability of this questionnaire was evaluated by Azadmarzabadi(21) using Cronbach’s alpha method and split-half technique in 2010 (0.78 and 0.65, respectively). The convergent and discriminant validity of the questionnaire with a confidence interval of 0.95 in all parts of the questionnaire was reported in 68.0%. The scores of questions in each item represent the value measured for each item with a range of 1 to 5 variations in which 1 is undesirable and 5 is desirable, and higher scores represent more health and safety in terms of stress. Total score ranges from 35 to 175. Demographic questionnaire and medical history questionnaires were used to determine the demographic characteristics and medical history of the subjects, respectively.

The experimental group (30 subjects) started exercise by doing three sessions, each lasting one hour, per week for a period of eight weeks under the planned exercise program. Each educational session included warm-up with stretching exercises for a 15-min period and aerobic exercise with moderate severity equal to 60% - 70% of the maximum heart rate. The exercise lasted 35 min, consisting of a set of movements, like stepping, walking, jogging, skipping, kicking, and arm swimming movements, performed to music. Thereafter, 10 min of cooling down consisted of stretching movements of muscles in lower extremities (especially quadriceps femoris muscles, hamstring muscles, gastrocnemius muscles, and gluteus muscles) and muscles in upper extremities and 1-min relaxation of whole body. During each session, five Polar pulse meters, as chest belt, were used randomly to observe the exercise severity for individuals moderately. he data collected were analyzed by using the Statistical Package for Social Science (SPSS) version 19. Descriptive analysis was used to describe the quantitative and qualitative variables of both groups; furthermore, analysis of variance with repeated measurements was used to compare changes in scores of the HSE occupational stress questionnaire.

## Results

**Diagram 1:**
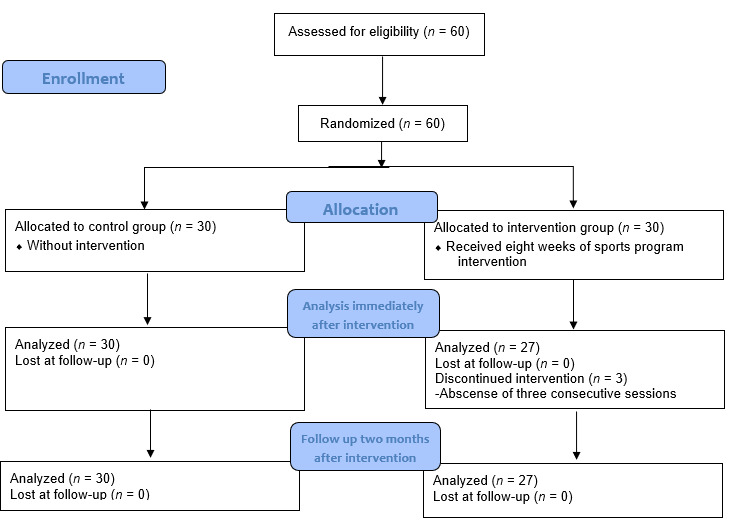
Flow chart

Diagram 1 shows that the 27 individuals from the intervention group vs. 30 from the control group were analyzed during the following moments: immediately and two months after the intervention. The cause of the three losses in the study group was their not attending for three consecutive times to the exercise program sessions.

Table 1 shows that no statistically significant difference existed in the general characteristics of the study groups. The characteristics of the whole group prevailed with age between 31 and 40 years (61.4%, mean of 33±2.7 years), married marital status (70.2%), with one to two children (56.1%), monthly income from 5 to 10-million rial (59.6%), official or A treaty employment (31.6% each), with work experience between 6 and 10 years (36.8%), and Bachelor educational level (93%).

**Table 1:** Comparison of demographic variables in control and intervention groups

		Group		
Variable	Control		Intervention	p value
	n (%)		n (%)	
Age				0.65
25-35	12 (40)		10 (37)	
31-40	18 (60)		17 (63)	
Marital status				0.24
Single	10 (33.3)		7 (25.9)	
Married	20 (66.7)		20 (74.1)	
Number of children				0.54
No children	10 (33.3)		6 (22.2)	
1-2 Children	17 (56.7)		15 (55.6)	
More than two children	3 (10)		6 (22.2)	
Monthly income				0.33
5-10-million rial*	19 (63.3)		15 (55.6)	
> 10-million rial	11 (36.7)		12 (44.4)	
Employment Status				0.21
Official	10 (33.3)		8 (29.6)	
A treaty	8 (26.7)		10 (37.0)	
Contractual	7 (23.3)		5 (18.5)	
Other	5 (16.7)		4 (14.8)	
Work Experience				0.32
1-5 years	9 (30)		10 (37)	
6-10 years	11 (36.7)		10 (37)	
> 10 years	10 (33.3)		7 ()	
Educational level				0.19
Associate Degree	1 (3.3)		1 (3.3)	
Bachelor	28		25 (93.3)	
Masters	1 (3.3)		1 (3.3)	

* 1 US Dollar = 42,105 rial

Table 2 shows that the HSE score in the control group was maintained in the three measurements. Rather, in the experimental group, the base HSE evaluation was equal to that of the control group, but immediately upon finishing the aerobic exercise program, the score was significantly higher than that of the control group (119.7 vs. 86.2). This score decreased after eight weeks of having finished the intervention, which, although higher than that found in the control group (91.4 vs. 85.8) this difference was not statistically significant. 

**Table 2:** Comparison of mean scores of occupational stress between both groups at various stages

Stage	Group	Mean ±SD	Mean Difference	t	DF	p value
Before intervention	Control	86.2±6.4	0.14	-0.062	55	0.95
	Experimental	86.3±5.7				
Immediately after intervention	Control	86.2±6.7	34.47	-10.39	55	<0.001
	Experimental	119.7±16.2				
Two months after intervention C	Control	85.8±6.5	5.85	-1.93	55	0.061
	Experimental	91.4±13.9				

## Discussion

The results, herein, demonstrated that the aerobic exercise program conducted for eight weeks is associated to diminished occupational stress, suggesting that this intervention should be kept over time. Among similar investigations, a study titled the “Effect of Regular Exercise on the Method of Coping with Problem-centered Stress in Nursing Students” was carried out by Dehghani *et al.*,(19) showed that by creating and protecting regular exercise behavior, we can enjoy the advantages of coping with stress and its negative outcomes, as well as its effect on the mental health of students. Another study on the effect of aerobic and non-aerobic exercise on the rate of anxiety by Purangbar *et al.,* (15) showed that anxiety in both groups of aerobic and non-aerobic exercise had significant decrease, compared with the control group. Therefore, we can conclude that exercise is an effective and safe way to reduce anxiety, and it seems that both aerobic and non-aerobic exercise could be effective in reducing anxiety. In addition, a study on reduction of pain and tension among hospital nurses after on-site massage treatments, carried out by Cooke *et al.*,(22) revealed that pain severity and tension was reduced significantly after the intervention.

The reason for such similarity may be the period of exercise, which was mostly eight weeks, and the type of samples who were mostly nurses. This could also be related to the physiological changes resulting from exercise activity. Physical exercise and activity cause the levels of some hormones to increase or decrease during exercise, compared with resting time. Catecholamines secreted from the central adrenal gland have a close relationship with the functions of Sympathetic Nervous System physiologically. Increased levels of Catecholamines are apparently important facilitators of exercise functions. Epinephrine and norepinephrine have various positive effects on cardio-vascular and metabolic systems of the body, considering their support role during exercise activities.(23)

The results of this research also showed that after eight weeks of aerobic exercise, a significant change in the occupational stress of nurses in the experimental and control groups was observed. This is an indication of the significant reduction of occupational stress after exercise and its subsequent increase two months after stopping exercise. 

The results of our research are in the same line with some studies(15,19, 22) that found the significant effect of exercise on stress and physiological changes of the body. For example, Abedian *et al*.(24) carried out a study to determine the effect of doing exercises on the rate of stress in midwives. In this study, the experimental group performed aerobic exercises at an intensity of 31% to 60% maximum oxygen consumption during 24 sessions for a period of 44 min, concluding that exercise reduces the rate of stress. In contrast with the results from our study, Ayatinasab *et al.*(25) studied the effect of aerobic and Yoga exercise on the self-efficacy of female employees of Sabzevar University of Medical Sciences and stated that aerobic exercises did not create any significant change in the self-efficacy of the samples, while Yoga exercise caused a significant change in the variable. The authors suggested that Yoga exercise caused more increment in the nervous system equilibrium compared with aerobic exercise.

The short period of doing exercise is among the limitations of the research and exercising for longer periods is suggested. By paying attention to the stressor nature of the nursing profession, it is recommended that the authorities highlight the health of this group of the society by taking measures to prevent problems in the work environment, which threatens their health. It is also necessary to suggest that nurses should consider the important results of this research, which shows the relationship between health and exercise, pay more attention to exercise activity, and add such activities to their daily program. 

Regular exercise could play an important role in improving the nurses’ mental health by influencing the coping strategies and reducing the negative outcomes of stress. Therefore, paying attention to aerobic exercise by nursing managers is recommended to reduce stress in nurses.
